# Small molecule and RNAi induced phenotype transition of expanded and primary colonic epithelial cells

**DOI:** 10.1038/srep12681

**Published:** 2015-07-30

**Authors:** Jutta Sharbati, Carlos Hanisch, Robert Pieper, Ralf Einspanier, Soroush Sharbati

**Affiliations:** 1 Institute of Veterinary-Biochemistry, Freie Universitaet Berlin, Oertzenweg 19 b, 14163 Berlin, Germany; 2 Institute of Animal Nutrition, Freie Universitaet Berlin, Koenigin-Luise-Str. 49, 14195 Berlin, Germany

## Abstract

Recent progress in mammalian intestinal epithelial cell culture led to novel concepts of tissue modeling. Especially the development of phenotypically stable cell lines from individual animals enables an investigation of distinct intestinal loci and disease states. We here report primary and prolonged culture of normal porcine epithelial cells from colon for cell line development. In addition, a novel primary three-dimensional intestinal culture system is presented, which generated organoids composed of a highly polarized epithelial layer lining a core of subepithelial tissue. Cellular characterization of monolayer cell lines revealed epithelial identity and pointed to a proliferative crypt cell phenotype. We evaluated both RNAi and chemical approaches to induce epithelial differentiation in generated cell lines by targeting promoters of epithelial to mesenchymal transition (EMT). By *in silico* prediction and ectopic expression, miR-147b was proven to be a potent trigger of intestinal epithelial cell differentiation. Our results outline an approach to generate phenotypically stable cell lines expanded from primary colonic epithelial cultures and demonstrate the relevance of miR-147b and chemical inhibitors for promoting epithelial differentiation features.

The intestinal epithelial monolayer consists of differentiated cells that constitute an interdependent organization with absorptive or secretory characteristics. The continuously self-renewing capacity of the intestinal epithelium, however, relies on the presence of less differentiated proliferating progenitor cells that emerge from intestinal stem cells. To date, it remains a challenge to mimic this highly organized system *in vitro* and basic research on intestinal epithelial biology requires the development of advanced cell culture models^1^. The high incidence of colon cancer arising from transformed colonic epithelial cells (CEC), pathological disorders such as inflammatory bowel diseases (IBD) as well as bacterial infections call for the development of adequate epithelial models, especially from the large intestine^2^.

Cell cultures generated by cellular extraction from the highly organized mucosal architecture lose the epithelial *in vivo* microenvironment. Consequently, cultured primary intestinal epithelial cells (IEC) potentially lack important regulatory components as it was demonstrated for the intestinal epithelial stem cell niche^3^. *In vitro* mimicking of expression signatures of the intestinal stem cell niche enabled cultivation and differentiation of intestinal stem cells^4^,^5^. A small proportion of matrix-embedded three-dimensional (3D) cells formed so-called organoids and differentiated into various cell lineages, thereby producing heterogeneous populations of both stem and differentiated cells. Consequently, modulation of the identified differentiation pathways might open up new possibilities for generation of differentiated IEC cultures *in vitro*^6^,^7^.

On that note, microRNAs (miRNAs) are recognized to be major regulators of cellular differentiation not only acting as intracellular modulators but also in a paracrine way via exosomes^8^,^9^. MiRNAs belong to the class of the non-coding RNAs that regulate gene expression via RNA interference (RNAi)^10^. Particularly as one given or a family of miRNAs is able to influence biological processes by interfering with multiple targets in specific signaling pathways^11^, we hypothesize that pathway modulation by means of either ectopic miRNA expression or inhibition can promote intestinal epithelial differentiation *in vitro*. For example, miR-200b has been shown to have multiple targets in EMT related pathways and to inhibit TGF-β1 induced EMT of intestinal epithelia, while upregulation of TGF-β1 was inversely correlated with miR-200b in IBD^12^. Furthermore, small molecule inhibitors (SMI) have also been shown to counteract TGF-β1 signaling antagonizing mesenchymal transition of renal tubular epithelial cells^13^. To address these issues, we aimed at developing a large intestinal epithelial cell culture system combining features such as low-passage numbers, sustained proliferative capacity and absence of transformation. We hypothesized that a series of successive enzymatic digestion steps of intestinal tissue can systematically produce epithelial cell populations from deeper crypt regions that harbor proliferative progenitor and stem cells.

## Results

### Primary and expanded colonic epithelial cell cultures

**Figure 1:**
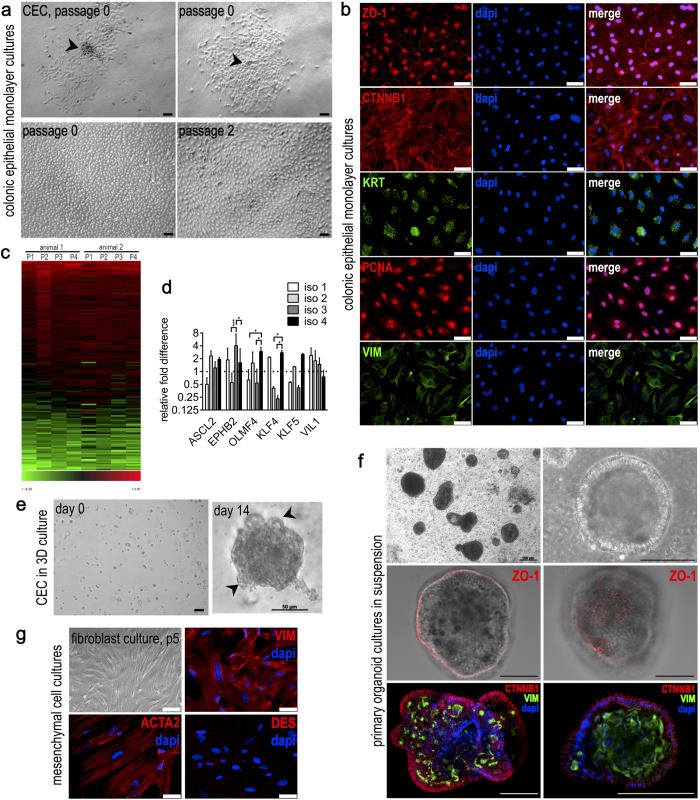
CEC maintain a stable phenotype and reflect progenitor features, while primary organoids reflect high degree of differentiation. (**a**) Phase contrast images show the outgrowth of CEC from microexplants (arrowheads) after 2d and 3d (upper panel). Primary and expanded CEC formed homogeneous monolayers as exemplified by microphotographs of passages 0 and 2 (lower panel). Scalebars 20 μm. (**b**) Immunostaining of CEC cultures for markers of epithelial differentiation, as demonstrated for tight junction protein ZO-1, adherens junction protein CTNNB1, and pan cytokeratin (KRT). PCNA staining was applied to indicate active proliferation. Mesenchymal traits are reflected by vimentin (VIM) expression. Scalebars 50 μm. (**c**) Stability of the CEC phenotype over 4 passages was evaluated by microarray analysis of gene expression. The heatmap reflects uniform gene expression across 4 passages (P1-P4) and of two independently sampled animals. Red and green colors indicate up- or down-regulation compared with native tissue (common reference). (**d**) RT-qPCR analysis of the intestinal stem cell markers *EPHB2, OLFM4* and *ASCL2* in CEC cell lines expanded from 4 different animals (iso 1 to 4). In addition, intestinal epithelial proliferation (*KLF4*) and differentiation markers *(KLF5, VIL1*) were quantified. Columns show means of three independent measurements, bars indicate standard deviations, asterisks summarize P values (*P < 0.05; **P < 0.01; ***P < 0.001). (**e**) Single cells derived from expanded CEC embedded in matrigel (day 0) formed 3D cultures, budding structures are indicated by arrowheads (day 14). Scalebar 20 μm or as indicated. (**f**) Primary intestinal organoids were maintained as suspension cultures (upper panel). Tight junctions (ZO-1) were detected close to the outer cell membranes of highly polarized epithelia and showed characteristic localization at cell junctions (middle panel). Adherens junctions (CTNNB1) were detected in the outer epithelial sheets of primary organoids, while VIM staining indicated the presence of fibroblasts in the subjacent tissue (lower panel). Scalebars 100 μm. (**g**) Mesenchymal monolayer cultures stained positive for alpha smooth muscle actin (ACTA2) as well as vimentin, while desmin was sparsely detected, pointing to a myofibroblast expression signature. Scalebars 50 μm.

For primary cell culture propagation, we developed a protocol, which included serial luminal digestion steps using collagenase and a subsequent mechanical disaggregation of the luminal tissue surface. Isolated cell suspensions were composed of a mixture of tissue fragments, cell clusters and single cells. IEC cultures mainly arose from microexplants of adherent multicellular structures. These yielded proliferating foci, gradually forming homogenous monolayers of highly proliferative cells (Fig. 1a). Accordingly, mucosa scrapings (fraction 6) were most efficient in formation of primary cultures. Cells isolated from younger individuals (11–26 d) possessed enhanced growth rates and plating efficiencies compared with animals aged 42–49 d.

### Expanded epithelial cell cultures display a crypt cell phenotype

Epithelial cultures from ascending colon were obtained by means of the protocol and provided the basis for the presented study. Primary and expanded CEC displayed a phenotypically uniform population with epithelial characteristics (Fig. 1a,b). Cells were actively proliferating and positive for PCNA staining (Fig. 1b). After passaging, fibroblast contaminations were typically not or only scarcely detectable as assessed by morphological features (Supplementary Fig. S1). For experimental approaches, we commonly used up to 15 passages. CEC exhibited various epithelial markers as demonstrated by immunofluorescence staining (Fig. 1b). The tight junction protein zona occludens-1 (ZO-1) displayed both junctional as well as nuclear staining, while adherens junction protein β-catenin (CTNNB1) was localized to the cell periphery (Fig. 1b). Cytokeratin (KRT) staining was observed in monolayer cultures, displaying both an intracytoplasmic and juxtanuclear expression pattern (Fig. 1b). A positive staining for vimentin (type III intermediate filament, VIM) was detected (Fig. 1b). Vimentin is both a marker for mesenchymal cells but also to used assess the degree of epithelial transition to a mesenchymal-like phenotype^14^.

### Impact of expansion on CEC phenotype and 3D culture

Morphological assessment showed that limited passaging had no obvious effect on CEC phenotype (Fig. 1a). For a fundamental verification of this observation, we performed a whole transcriptome microarray study using isolates from 2 different animals and CEC culture passages 1 to 4. Figure 1c shows a representative cluster of 160 genes, which were robustly expressed in all studied samples. Data analysis did not reveal any effect of subcultivation on global gene expression. When comparing two different isolates, interindividual effects on expression profiles were not observed. Functional annotation of genes showing robust expression in cell culture (log2 ratios ≥0 compared with native tissue) pointed to metabolic activity, migratory capacity and a stable degree of mesenchymal marker expression, thereby supporting the observed crypt-like character.

The proliferative and crypt-like phenotype of CEC monolayers prompted us to analyze the expression of marker genes of intestinal epithelial stem cells (IESC). Expression of *EPHB2, OLFM4* and *ASCL2*^15^,^16^,^17^ was verified in low passage CEC cultures from four independent isolates (Fig. 1d), while *LGR5*^4^ expression was not detected (data not shown). In addition, we quantified gene expression of markers of *in vivo* proliferation or differentiation. It is known that *in vivo* Krüppel-like factor (KLF) 4 is expressed in terminally differentiated epithelial cells at the villus borders of the mucosa, while KLF5 is localized to epithelial cells at the base of intestinal crypts^18^. Villin (VIL1) is associated with microvilli of differentiated epithelia^19^. Both proliferation and differentiation markers were expressed in all CEC cell cultures. Although some genes exhibited significantly different expression among isolates, there was no systematic pattern observed (Fig. 1d). Based on these observations, we asked if isolated CEC are capable of forming 3D structures using cell culture conditions that have been described to maintain stem cell features^4^. Using single cell suspensions embedded in a 3D matrix, we promoted growth of multicellular structures. A small proportion of individual cells (about 1%) were able to proliferate under these conditions. The efficiency is comparable to published colony-forming efficiencies (below 1%) of single sorted LGR5^+^ small intestinal stem cells^4^. Budding structures were observed at the periphery (Fig. 1e).

### Intestinal organoid cultures of highly polarized epithelia lining a mesenchymal core

Using the described protocol, we observed the formation of primary intestinal organoids in the supernatants of primary intestinal monolayer cell cultures (Fig. 1f). The organoids were maintained without the use of a matrix as suspension cultures. Viable organoids were observed for at least two weeks as exemplified by microphotographs of representative organoids up to day 16 (Supplementary Fig. S2). Tight junction immunostaining (ZO-1) revealed an apical localization closely related to outer epithelial membranes, while CTNNB1 immunostaining demonstrated the presence of adherens junctions (Fig. 1f). Subjacent of the basal epithelial membranes, we observed a core of connective tissue of mostly mesenchymal origin, as indicated by vimentin (VIM) staining. Organoid cultures were composed of both round as well as irregular shaped structures (Fig. 1f).

### Associated isolation of intestinal myofibroblasts from individual animals

After epithelial cell isolation, the remaining tissue was used to set up mesenchymal cell cultures. These cultured cells were spindle-shaped and positive for α smooth muscle actin (ACTA2) and vimentin (VIM), but devoid of desmin (DES) expression (Fig. 1g). This expression pattern is consistent with current definitions of the intestinal myofibroblast phenotype^20^,^21^.

### SMIs drive epithelial differentiation of CEC counteracting TGF-β1

**Figure 2:**
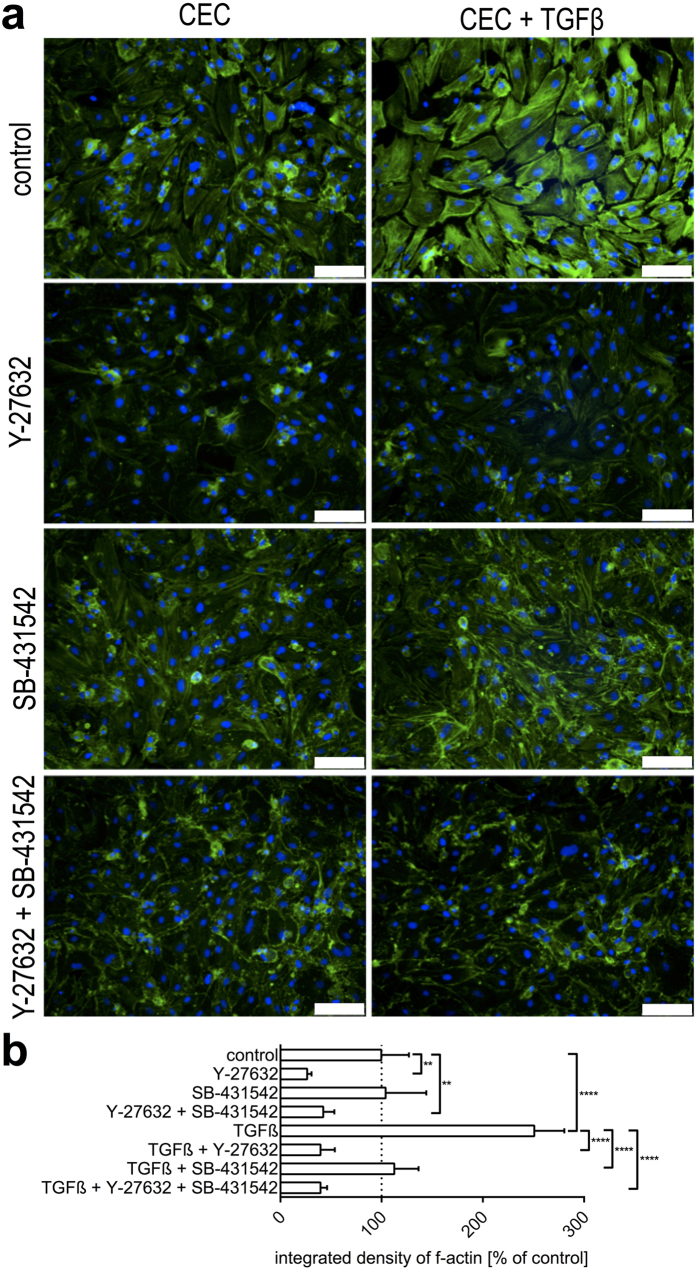
Small molecule inhibitors suppress EMT. Both Y-27632 (ROCK inhibitor) and SB-431542 (TGF-β1 type I receptor kinase inhibitor) as well as a combination thereof were tested to counteract EMT employing CEC as a model. (**a**) Actin reorganization was demonstrated by fluorescent F-actin staining (phalloidin, green). Control cells showed both stress fibers and cortical actin staining, while TGF-β1 treatment potently induced actin stress fiber formation and elongated morphology. Treatment with Y-27632 diminished stress fiber formation and enhanced cortical actin staining in untreated cells as well as after experimental EMT induction by TGF-β1. SB-431542 supplementation did not affect stress fiber formation distinctly in untreated cells, but enhanced cortical staining in TGF-β1 treated CEC. A combinatory application of both chemicals potentiated observed effects and markedly reduced EMT associated characteristics. Scalebars, 50 μm. (**b**) Quantification of total actin staining compared with nontreated controls was performed using ImageJ software. Both single treatment with Y-27632, as well as a combination of Y-27632 and SB-431542 significantly reduced total actin staining. TGF-β1 induced EMT was antagonized by treatment with either Y-27632, SB-431542 or a combination of both as indicated by significant reduction of total actin signals. Asterisks indicate P values (*P < 0.05; **P < 0.01; ***P < 0.001; ****P < 0.0001).

We employed SMI to promote pathways affecting CEC phenotype transitions. In a study using renal tubular epithelial cells, Das *et al*. demonstrated that SB-431542 (inhibitor of TGF-β1 type I ALK receptors) and Y-27632 (inhibitor of Rho-kinases ROCK1 and 2) antagonized TGF-β1 induced mesenchymal transition^13^. We conferred these findings to our study by supplementation of CEC cultures with each SMI or a combination of both, and assessed actin reorganization of native CEC or after TGF-β1 mediated EMT induction (Fig. 2). After 24 h incubation on CEC monolayers, Y-27632 eliminated stress fibers and led to a localization of actin to the cell periphery (Fig. 2a). SB-431542 did not affect localization of actin distinctly compared with non-treated controls. However, a combination of both chemicals markedly increased cortical actin bordering. We then tested if these chemicals have the potential to prevent TGF-β1 induced EMT. In TGF-β1 treated cells, Y-27632 led to a strong reduction of stress fiber formation, and SB-431542 partly inhibited EMT induction, as cortical actin staining increased compared with TGF-β1 induced controls. Again, the combination of SB-431542 and Y-27632 proved most potent in prevention of TGF-β1 induced EMT, as the cell size was decreased, non-cortical actin filaments were mostly absent and intercellular adhesion disassembly was prevented (Fig. 2a). Quantification of total fluorescence intensities demonstrated significantly decreased actin staining both after treatment with Y-27632 or a combination of SB-431542 and Y-27632 compared with untreated controls (Fig. 2b), while SB-431542 treatment alone did not have an impact. After TGF-β1 induced EMT, single treatment with SB-431542 and Y-27632 and a combination thereof led to significantly reduced actin staining (Fig. 2b).

### Impact of microRNAs on CEC phenotype

**Figure 3:**
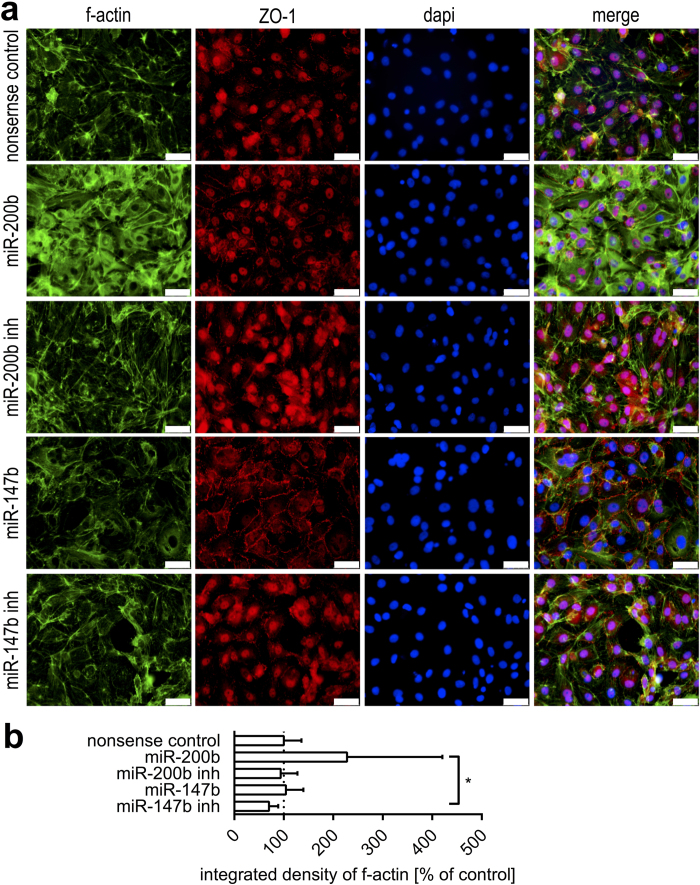
miRNA mimics and inhibitors affect CEC phenotype. (**a**) The potential of miRNAs to direct CEC differentiation features was evaluated by fluorescent staining of tight junction protein ZO-1 (red) and f-actin (phalloidin, green). MiR-200b was shown to drive CEC differentiation, as evidenced by enhanced ZO-1 staining, while the miR-200b inhibitor had the opposite effect. MiR-147b caused pronounced ZO-1 localization to cell borders and reduction of stress fibers. MiR-147b diminished nuclear ZO-1 staining compared with all other treatments or nonsense controls. The inhibition of intrinsic miR-147b markedly reduced epithelial differentiation features, as demonstrated by reduced cell-cell adhesion. Scalebars, 50 μm. (**b**) Total actin signals were quantified using ImageJ software. No statistically significant changes of total actin signals were observed comparing transfected cells to nonsense transfected controls. Asterisks indicate P values (*P < 0.05).

MiRNAs are known to control cell fate decisions and differentiation of IEC. In this context, Chen *et al*. demonstrated that miR-200b inhibited TGF-β1 induced EMT in a small intestinal cell line^12^. Therefore, we used miR-200b mimic and inhibitor to assess responsiveness of CEC regarding phenotype transition. We assessed intercellular adhesion, actin remodeling and cellular migration as important hallmarks of EMT. As shown in Fig. 3a, the transfection of miR-200b mimic led to a reduction of mesenchymal traits as displayed by a more cuboidal cell shape and actin reorganization, while total actin staining tended to increase due to diffuse cytoplasmic staining (Fig. 3a,b). Inhibition of miR-200b induced mesenchymal characteristics as judged by spindle cell morphology induction, stress fiber formation and reduced tight junction formation (Fig. 3a). Using a wound-healing assay, cell migration was assessed 21 hours after creating a cell free gap by quantifying the degree of gap closure (Supplementary Fig. S3). Cell migration appeared reduced after miR-200b inhibition (69 ± 29% gap closure) compared with miR-200b mimic transfection (93 ± 13% gap closure) or nonsense transfected controls (81 ± 7% gap closure), however, these differences were not statistically significant. Since miR-195 was reported to influence migratory capacities of an intestinal epithelial cell line^22^, we additionally assessed effects of miR-195 and its paralogue miR-497 on tight junction formation and actin remodeling, however, no effects on CEC phenotype were detected (data not shown).

### miR-147b is a novel regulator of differentiation in normal CEC antagonizing EMT

We focused on identifying novel potential regulators of CEC phenotype by searching for miRNAs being able to target multiple EMT triggering genes. To achieve this, we performed a miRmap analysis^23^ and considered highly scored miRNAs (miRmap score ≥90) targeting following EMT factors: *ZEB1/2, SNAI1/2/3, SMAD2/3/4, TWIST1* and *VIM*. After intersecting the target lists, we identified miR-147b as a highly conserved molecule targeting *ZEB1/2, SMAD2/3/4* as well as *SNAI2/3* both in humans and pigs. Pathway analysis of potential miR-147b targets was performed as described earlier (Bohmer *et al*., 2013), and pointed out its EMT inhibitory potential (data not shown).

Upon transfection of CEC with miR-147b mimics, the actin cytoskeleton underwent profound remodeling, as visualized by f-actin staining (Fig. 3a). In control cells, actin staining localized to the cell cortex as well as to stress fibers and focal adhesions. In miR-147b transfected CEC, stress fibers mostly disappeared and cells displayed a pronounced cuboidal and regular morphology (Fig. 3a). At the plasma membrane, staining of tight junction protein ZO-1 markedly increased and appeared more continuous. Furthermore, miR-147b led to reduced ZO-1 signals located to the nucleus. In contrast, miR-147b inhibition led to a strong induction of actin staining localized to stress fibers and focal adhesions and reduced staining at the cell cortex. Tight junction staining markedly decreased, while nuclear staining was enhanced after miR-147b inhibition (Fig. 3a). Apart from observed changes in actin localization, quantification of total actin signal intensities did not show significant differences between transfected cells (Fig. 3b). Furthermore, miR-147b tended to accelerate migration of CEC (100% gap closure after 21 h) compared with both miR-147b inhibition (85 ± 19%) and nonsense controls (81 ± 7% gap closure) (Supplementary Fig. S3). However, effects on migratory behavior were not statistically significant.

## Discussion

Cell culture generation from large intestine is of particular interest for disease-related or toxicological studies, as the colon is most susceptible to cancer or inflammatory diseases. In this study, we developed an approach that reliably produces expandable CEC cultures. CEC are efficiently isolated and grown *in vitro* by targeting intestinal epithelial crypt cells and maintaining some degree of mutual cell contacts as well as contacts to extracellular matrix. The presented protocol has the advantage that only a limited number of animals is required for primary culture generation, which is successively expanded for functional approaches. In addition, we integrated propagation of mesenchymal cell cultures into our approach. This facilitates the study of mesenchymal contribution to intestinal homeostasis and disease, as e.g. myofibroblasts adjacent to crypt epithelia are discussed as sources of Wnt-ligands *in vitro*, and co-cultures are effective in promoting crypt organoid growth^24^. On a different note, piglets constitute an appropriate donor for IEC isolation since they resemble human intestinal biology very closely. Because of their close relation to humans in terms of anatomy, genetics and physiology they were suggested to represent a valuable model for e.g. human infectious diseases^25^.

As our general aim for epithelial cell culture generation was to maintain a proliferative and stable phenotype, terminal differentiation of CEC was inherently not observed under the cell culture conditions applied. Beyond the scope of this study, it would be highly desirable to define means for *in vitro* manipulation of differentiation in the context of intestinal disease models. However, mechanisms involving intestinal epithelial differentiation and polarity are only beginning to be unveiled and translated into cell culture techniques^6^. In this context, we further characterized the proliferative crypt cell phenotype of CEC cultures. We verified intestinal stem cell marker expression and observed formation of 3D cultures in conditions promoting stem cell expansion^4^, although we did not detect *LGR5* expression. This indicates that CEC cultures have progenitor features and might reflect fast growing transit cells that are located in the transit amplifying zone of colon crypts *in vivo*, but do not contain stem cell populations. Alternatively, CEC cultures *in vitro* may have descended from stem cells that reside in microexplants. Also, the applied cell culture environment could have imposed a degree of mesenchymal transition, which promoted the observed phenotype, as several studies revealed a link between EMT and stemness of cells^26^. It is known that TGF-β1 signaling plays an important role in EMT, while Rho-kinases are key regulators of the cytoskeleton and cell polarity^27^,^28^. A study using primary cell cultures from renal tubular epithelia demonstrated that chemical inhibition of these pathways reversed an experimentally induced mesenchymal transition^13^. In line with this, we evaluated effects of supplementation with SMI of TGF-β1 type I ALK receptors and Rho-kinase, SB-431542 and Y-27632 respectively. Using a combination of both chemicals in short term supplementation, we observed profound reorganization of the actin cytoskeleton and reversal of TGF-β1 induced EMT in CEC cultures.

Our recent studies have shown that one particular miRNA is capable of regulating a given pathway by targeting several members of a signaling cascade^11^,^29^. Based on this knowledge, we predicted and verified miR-147b as a conserved molecule that potently induces epithelial differentiation features, as demonstrated by profound modulation of the actin cytoskeleton and markedly increased tight junction detection. We additionally observed a decrease of nuclear ZO-1 localization mediated by miR-147b. In the literature, a nuclear localization of ZO-1 has been well documented^30^,^31^. ZO proteins possess several nuclear localization (NLS) as well as nuclear export signal (NES) domains, enabling them to shuttle between the cytoplasm and the nucleus. ZO-1 was described to interact with nuclear proteins as well as with dual residency (cytoplasmic/nuclear) proteins like ZONAB, which binds to promotor regions of PCNA and regulates epithelial cell proliferation^32^. *In vivo*, PCNA protein expression of porcine intestinal epithelial cells is restricted to the crypt compartment of proliferative stem and transit amplifying cells, while differentiated epithelia are mostly devoid of PCNA staining (Supplementary Fig. S4). CEC cultures exhibited distinct nuclear PCNA staining as well as nuclear and junctional localization of ZO-1 (Fig. 1b). Taken together, we assume that CEC cultures reflect an intermediate state between proliferative crypt and differentiated epithelial cells.

In line with our study, Lee *et al*. applied colon cancer cell lines to study the role and potency of miR-147 for the reversal of EMT^33^ and proposed a tumor suppressor function of miR-147. Interestingly, Lee *et al*. observed inhibited proliferation by miR-147 in cancer cell lines. On a different note, many studies describe inhibited proliferation, migration and invasion of cancer cells by miR-200b^34^,^35^. In contrast, based on using the non-transformed small intestinal cell line IEC-6, Chen *et al*. verified miR-200b to promote rather than to inhibit cellular growth by increasing S-phase entry and enhancing expression of the protein cyclin D1^12^. Along these lines, we reported an increased migratory capacity by both EMT inhibiting miRNAs miR-147b and miR-200b in CEC cultures, although effects were not statistically significant. These contrary observations in normal vs. cancer cells point out that miRNAs are key regulators of epithelial homeostasis and exert different roles in cellular health and disease conditions. Moreover, these observations reflect that non-transformed cell models might better represent normal intestinal epithelial physiology.

In addition, the introduced primary colonic organoid cultures are a novel tool providing an advanced biological complexity as they contain a mesenchymal core that is lined with differentiated epithelial cells. On one hand, this kind of organoid culture obviates the need for matrix-based cultivation using hydrogels. On the other hand, the highly polarized epithelial cytoarchitecture is preserved and the apical epithelial membrane is oriented to the culture medium. This provides an advanced accessibility for experimental manipulation or for mimicking the intestinal exposure e.g. to pathogenic challenges.

In conclusion, we established organoid cultures as a model, which structurally and morphologically resembles the intestinal epithelial architecture *in vivo*. On the other hand, we presented comprehensive evidence of the suitability of CEC cultures for mechanistic studies, as CEC are highly flexible low passage cell lines that can readily be used for functional studies employing RNAi or small molecule based modulation of genes of interest. As a future perspective, it would be compelling to apply organoid cultures for targeted hypothesis generation in diverse intestinal research areas. In turn, CEC cultures may be applied for exploration of mechanisms underlying the experimental data generated in organoids. In addition, future studies may identify culturing conditions, which allow an evaluation of self-renewal and proliferation of potential crypt cells in organoid cultures. Due to the high incidence of gastrointestinal disorders, comprehensive and individual culture systems are needed for studying gut pathologies. In this line, we propose a combinatory approach of two distinct intestinal cell culture models, while the novel insights about SMI and miRNA-induced epithelial phenotype transitions contribute to the understanding of intestinal epithelial signaling.

## Methods

### Animals and collection of intestinal tissues

All animal experiments were approved by the local state office of occupational health and technical safety ‘Landesamt für Gesundheit und Soziales Berlin’ (LaGeSo Reg. Nr. 0347/09) and carried out in accordance with the approved guidelines. Intestinal tissue was taken from 24 purebred Landrace piglets at the age between 11 and 49 days. The piglets were sedated with 20 mg/kg bodyweight (BW) of ketamine hydrochloride (Ursotamin, Serumwerk Bernburg AG) and 2 mg/kg BW of azaperone (Stresnil, Jansen-Cilag) prior to euthanasia with intracardial injection of 10 mg/kg BW of tetracaine hydrochloride, mebezonium iodide and embutramide (T61, Intervet). Tissue was collected from ascending colon (approximately 5–10 cm each).

### Cell isolation and culture

For the optimized cell isolation protocol, the lumen of intestinal sections was successively flushed using 300 ml warm (37 °C) wash buffer and wash medium. Tissues were transported to the laboratory in transport medium 1 (37 °C), while the lumen was filled with transport medium 2. Subsequently, isolation procedures for epithelial cells were initiated within 30–90 min. For enzymatic digestion, the intestinal lumen was filled with warm (37 °C) medium containing 1 mg/ml collagenase (Biochrom) and four successive incubation steps (30 min each) were performed. Cell suspensions were collected after each incubation step (fraction 1 to 4), centrifuged for 7 min at 100 × g, and suspended in culture medium V1 (Supplementary Table S1). The lumen was gently squeezed and released cells were suspended in culture medium (fraction 5). Finally, the lumen was opened and the mucosa was gently scraped off and suspended in 5 ml culture medium V1 (fraction 6). All cell suspensions consisted of both single cells and small tissue fragments. The fractions were further diluted in culture medium V1 and plated in 25 cm^2^ cell culture flasks (Corning CellBIND surface). Medium was first changed after 24–72 h. Cells were cultivated in a 37 °C humidified incubator in a 5% CO_2_ atmosphere, medium was changed every 2 or 3 days at 80–90% confluence and cells were split at a ratio of 1:4 to 1:6 using accutase (Sigma). After 2 subcultivations, culture medium V2 was applied for epithelial cell cultures. Cultured cells were cryopreserved using Cryo Maxx S (PAA). For primary intestinal organoid cultures from ascending and transverse colon 1 mg/ml collagenase type IA (Sigma) was used, applying an otherwise identical protocol as above. Organoid cultures were collected from supernatants of primary monolayer cultures that were obtained using fraction 6. Primary organoids were maintained as suspension cultures and 50% of the culture medium V1 was replaced twice weekly.

For mesenchymal cell cultures, tissue explant-outgrowth was performed using small fragments (~4 mm^2^) of the luminal surface of intestinal sections after the enzymatic digestion protocol described above. Cell culture propagation required fibroblast culture medium and standard 75 cm^2^ cell culture flasks (Sarstedt). Composition of all buffers and media are described in Supplementary Tables S1 and S2.

### Three-dimensional CEC culture in a matrix

Low passages of cultured intestinal cells (passage 2 to 5) were detached in order to produce single cell suspensions. 5000 cells were embedded in 30 μl Matrigel using 96 well plates (Matrigel GF-reduced, BD Biosciences, protocol adapted from Sato *et al*.^4^). The matrix-cell suspension was placed in a 37 °C incubator to allow the matrix to solidify. Finally, the matrix was overlaid with 200 μl 3D culture medium (Supplementary Table S2).

### Immunofluorescence staining

For paraffin embedded tissues a standard rehydration protocol for 10 μm sections was applied as described earlier^36^. If necessary, antigen retrieval was achieved by boiling in 10 mM citrate buffer pH 6 for 40 min. Non-specific binding of primary antibodies was blocked by incubation in PBS with 10% serum of the species the secondary antibody was raised in for 1 h at room temperature. The antibodies and further experimental conditions used for immunofluorescence staining are listed in Supplementary Tables S3 and S4. Nuclei were counterstained using 200 ng/ml 4′, 6-Diamidin-2-phenylindol (DAPI, Roche) in PBS for 5 min, and sections were embedded in 50% glycerol in PBS.

Intestinal cell cultures were grown on 8-well tissue culture chambers (Sarstedt) or μ-Plates (Ibidi). Conditions for fixation, permeabilization and staining are described in electronic supplementary material, Table S3. Blocking was performed using 1% BSA in PBS for 1 h at room temperature. Counterstaining and embedding was performed as mentioned above. Phalloidin atto-488 (Sigma) was used for actin staining according to the manufacturer’s instructions. Images were acquired using a Leica DMI6000B inverted microscope and Leica LAS-AF software or a Zeiss Axiovert 25 inverted microscope. Quantitative analysis using raw images from immunofluorescence stainings of replicate experiments was performed using ImageJ^37^.

### Microarray experiments and RT-qPCR

Total RNA from cultured cells was isolated using the mirVana miRNA Isolation Kit (Ambion) and quality was controlled as described previously^38^. For this purpose, intestinal cells (passage 1–4) originally isolated from ascending colon of 2 individual piglets were cultivated in 25 cm^2^ cell culture flasks until confluence. Additionally, RNA isolated from colon tissue was used as a common reference. Microarray analysis was performed as described previously^11^ using a swine protein-annotated 70-mer oligonucleotide microarray representing 20,400 *Sus scrofa* ESTs. MIAME-compliant data of all performed microarrays considering the applied platform as well as processed and raw sample data were submitted to the NCBI’s Gene Expression Omnibus^39^ and are accessible through GEO Series accession number GSE55739. Data analysis was performed as described earlier^40^. Quantification of mRNA expression by means of RT-qPCR was performed as described earlier^40^. Oligonucleotides used in this study are listed in Supplementary Table S5.

### SMI treatment

We treated the cell cultures with 2 ng/ml TGF-β1 (R&D) for 48 h (24 h after seeding of cells), 5 μM or 50 μM of SB431542 (Sigma); 1 μM or 10 μM of Y27632 dihydrochloride (abcam) as indicated.

### Transfection

For transfection studies CEC (up to passage 15) were cultured in DMEM/Ham’s F12 (1:1), 5% FCS and 10 μg/ml gentamicin. Amaxa Basic Nucleofector Kit for Primary Mammalian Epithelial Cells (Lonza) was applied according to the manufacturer’s instructions using 0.5 × 10^6^ cells per sample. Replicate experiments were performed using cells originating from 2 different animals (aged 11 and 26 d) in order to account for inter-individual variation. For miRNA studies mimics and inhibitors were used for following miRNAs: miR-200b, miR-147b, miR-195, miR-497 (30 pmol per sample). Nonsense miRNA Pre-miR miRNA Precursor Negative Control #1 served as a negative control, Cy3 Dye-Labeled Pre-miR Negative Control #1 was used to determine transfection efficiency (all from Life Technologies).

### Wound healing assay

To profile chemical or miRNA induced modulation of the epithelial phenotype, we cultured the cells in DMEM/Ham’s F12 (1:1), 5% FCS and 10 μg/ml gentamicin. For migration assessment, we applied silicone cell culture inserts (Ibidi) into 24 well plates (CellBIND, Corning). We seeded 3 × 10^4^ cells in each well of ibidi inserts, and removed the inserts after 24 h of cultivation, thereby producing a cell free gap of 500 ± 50 μm that was observed for assessment of migratory behavior. For analysis, we assessed eight independent replicates.

### Statistical analysis

Statistical analysis of RT-qPCR as well as migration assay data was performed using ordinary two-way ANOVA with Tukey’s multiple comparisons test. The statistical comparison of quantitative immunofluorescence data was performed using one-way ANOVA with Holm’s-Sidak’s multiple comparisons test with a single pooled variance. All tests were conducted applying GraphPad Prism version 6.00 for Windows, GraphPad Software, La Jolla California USA, www.graphpad.com. Asterisks in figures summarize P values (*P < 0.05; **P < 0.01; ***P < 0.001; ****P < 0.0001).

## Additional Information

**How to cite this article**: Sharbati, J. *et al*. Small molecule and RNAi induced phenotype transition of expanded and primary colonic epithelial cells. *Sci. Rep*. **5**, 12681; doi: 10.1038/srep12681 (2015).

## Supplementary Material

Supplementary Information
