# Supplementary material for: Small molecule and RNAi induced phenotype transition of expanded and primary colonic epithelial cells
Source: Sci Rep. 2015 Jul 30;5:12681. doi: 10.1038/srep12681 (PMC4519788; doi:10.1038/srep12681)
Supplement: Supplementary Information [file srep12681-s1.pdf]

Small molecule and RNAi mediated phenotype transition of primary and expanded colonic epithelial cells

Jutta Sharbati, Carlos Hanisch, Robert Pieper, Ralf Einspanier, Soroush Sharbati

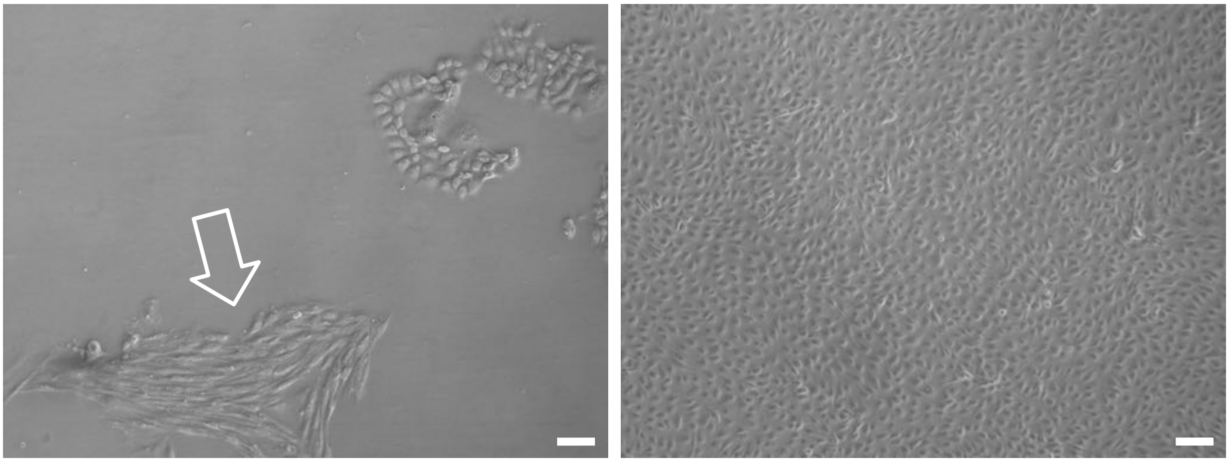

Supplementary Figure S1: The arrow shows rarely appearing fibroblast contaminations, while right figure shows homogenous CEC culture from same animal. Both figures represent cultures isolated from a 26 d old piglet at passage 0 on day 2 after seeding. Cultures with fibroblast contaminations were discarded. Scale bars represent 20 µm.

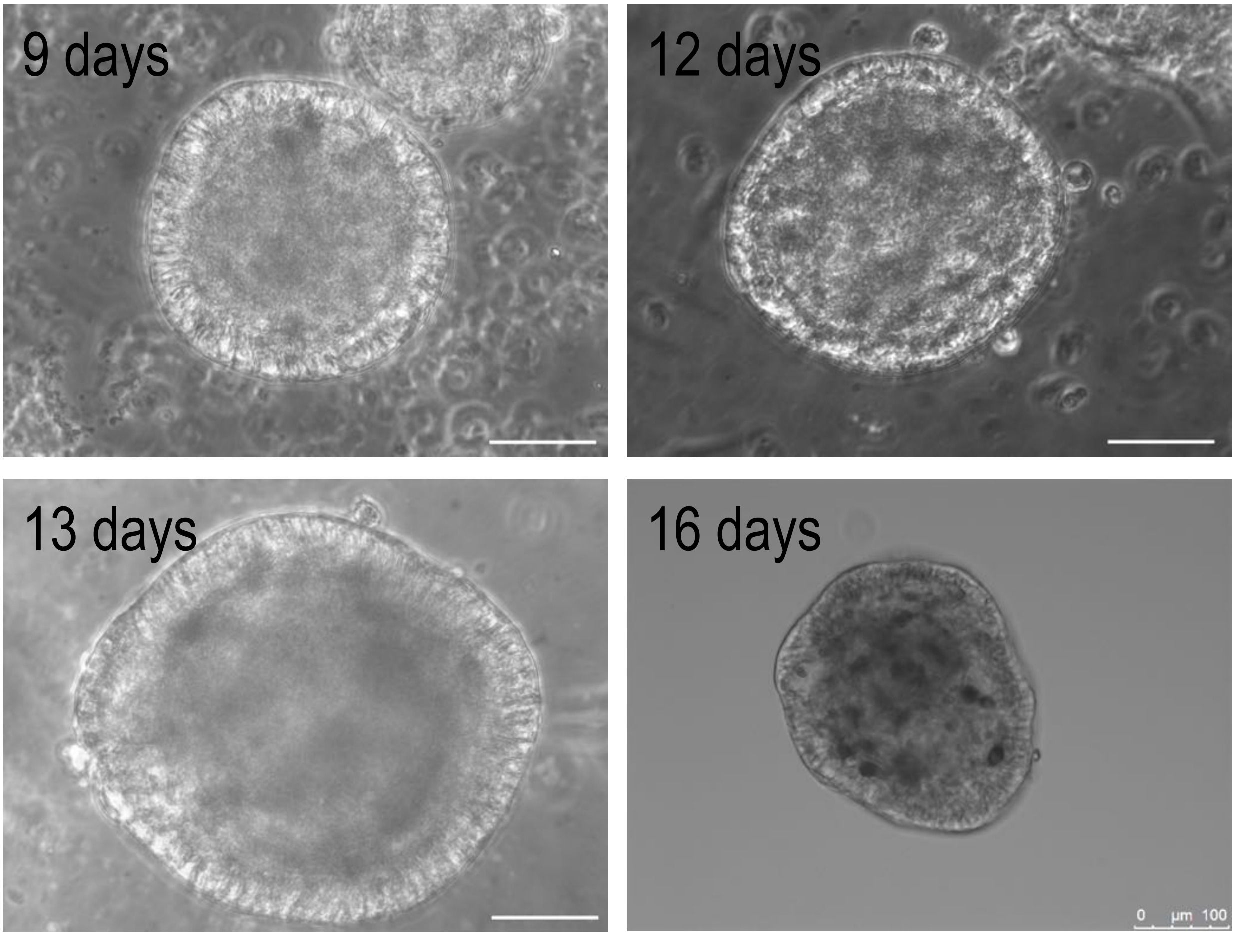

Supplementary Figure S2: Microphotographs of different organoids in suspension. The organoid architecture was composed of a mesenchymal core lined with highly differentiated epithelial cells and remained intact in suspension culture over extended periods of time. Scale bars represent 100 µm.

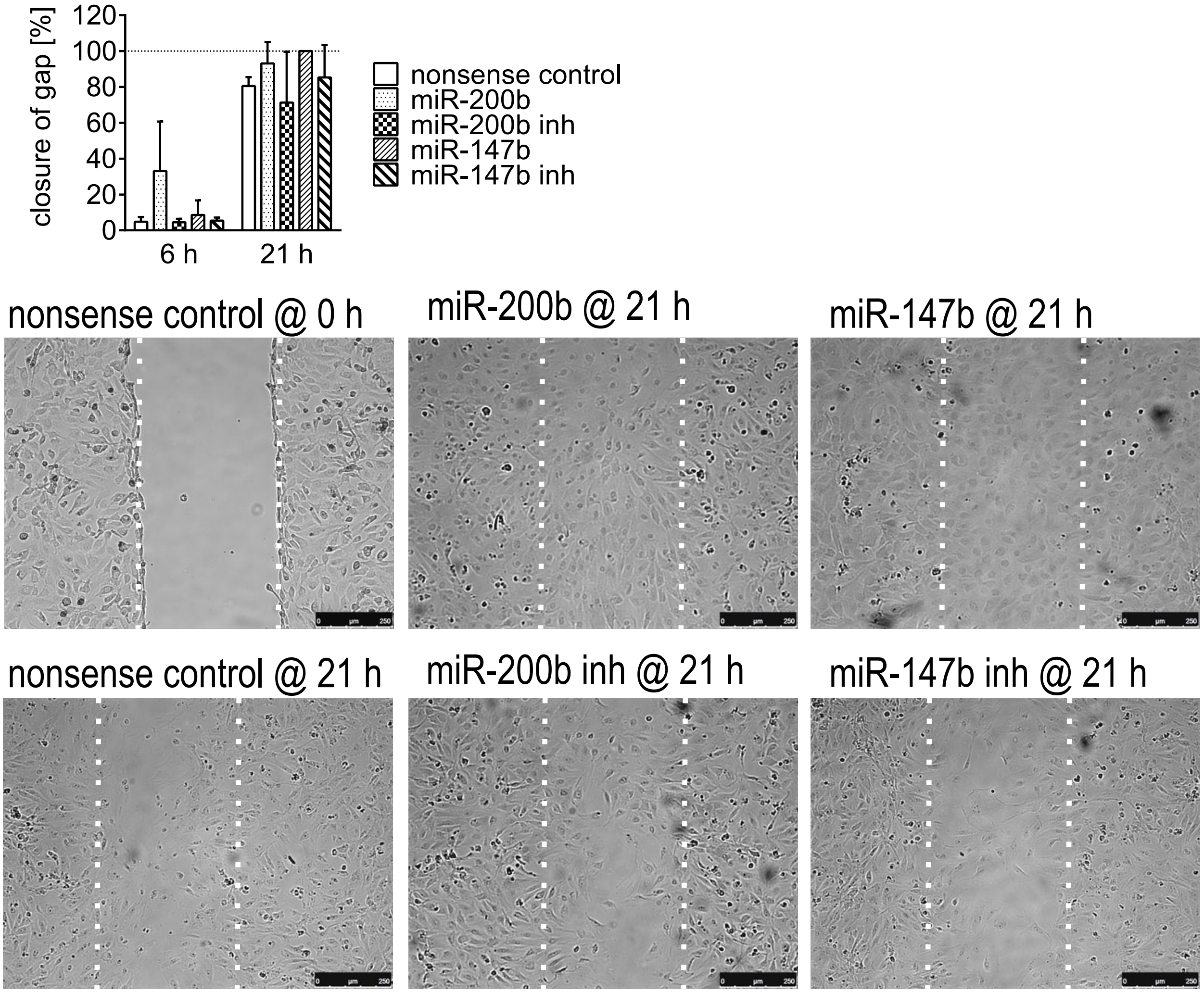

Supplementary Figure S3: The bar diagram shows mean percentage of gap closure at 6 and 21 h after removing the insert (barrier). Ectopic miR-200b and miR-147b caused enhanced migration while respective inhibitors had no obvious effects (P > 0.05). Representative microphotographs show nonsense controls at 0 h (initial gap after removing the barrier) and migration of CEC after indicated treatments at 21 h. Initial gaps are indicated by dotted lines. Scale bars represent 250 µm.

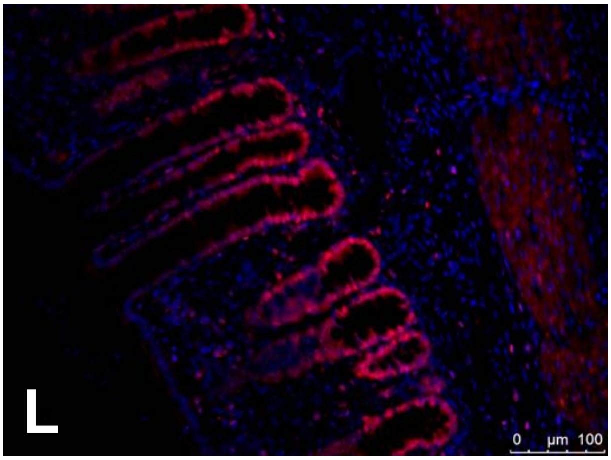

Supplementary Figure S4: Immunofluorescence PCNA (red) staining of paraffin-embedded sections of porcine colon counterstained with dapi (blue). Actively proliferating cells positive for PCNA were predominantly located at the bottom of crypts and PCNA expression apparently decreased along the crypt axis to the lumen (L).

Supplementary Table S1

Supplement	Wash Buffer	Wash Medium	Transport Medium 1	Transport Medium 2	Culture medium V1	Culture medium V2	Fibroblast culture medium
**Basal medium**	HBSS (Biochrom)	DMEM/Ham's F12 1:1 (Biochrom)	DMEM/Ham's F12 1:1 (Biochrom)	DMEM/Ham's F12 1:1 (Biochrom)	DMEM/Ham's F12 1:1 (Biochrom)	DMEM/Ham's F12 1:1 (Biochrom)	DMEM (Biochrom) 1g/l D-glucose
**Fibroblast-conditioned DMEM medium**	-	-	-	-	1/3	1/3	-
**Gentamicin (Biochrom)**	10 µg/ml	10 µg/ml	10 µg/ml	10 µg/ml	10 µg/ml	10 µg/ml	10 µg/ml
**Amphotericin B (Biochrom)**	0.5 µg/ml	0.5 µg/ml	0.25 µg/ml	0.25 µg/ml	0.25 µg/ml	-	0.25 µg/ml
**Penicillin/ Streptomycin (Biochrom)**	200 U/ml, 200 µg/ml	200 U/ml, 200 µg/ml	100 U/ml, 100 µg/ml	100 U/ml, 100 µg/ml	100 U/ml, 100 µg/ml	-	100 U/ml, 100 µg/ml
**NEA (100x) (Non-essential amino acids, Biochrom)**	-	-	-	-	1x	1x	-
**ITS (100x) (Insulin-transferrin-sodium selenite media supplement, Sigma)**	-	-	-	-	1x	-	-
**Heparin (Biochrom)**	-	-	-	-	9 U/ml	9 U/ml	-
**EGF 0.1 mg/ml sigma E1257**	-	-	-	-	0.01 µg/ml	0.01 µg/ml	-
**Ascorbic acid (Sigma)**	-	-	-	10 µg/ml	10 µg/ml	10 µg/ml	-
**Glutathione (Sigma)**	-	-	-	10 µg/ml	10 µg/ml	10 µg/ml	-
**Serum (FBS superior, Biochrom)**	-	-	-	-	5 %	5 %	10 %

Supplementary Table S2

Supplement	3D Culture Medium
**Basal medium**	Advanced DMEM/Ham's F12 1:1 (Life Technologies)
**Gentamicin (Biochrom)**	10 µg/ml
**EGF 0.1 mg/ml sigma E1257**	50 ng/ml
**HEPES (Biochrom)**	10 mmol/l
**Glutamax**	1x
**N2**	1x
**B27**	1x
**N-Acetylcysteine**	1 µM
**Noggin(R&D)**	50 ng/ml
**R-Spo-1 (R&D)**	500 ng/ml
**Wnt3a(R&D)**	25 ng/ml
**Nicotineamide**	10 mM

Supplementary Table S3

Primary Antibody	Fixation	Company	Permeabilisation	Concentration of Stock Solution	pAb working dilution
**Pan Cytokeratin**	Acetone; -20 °C, 20 min	Dako Clone AE1/AE3	-	Not specified	1:50
**ZO-1**	Histofix 4 % (Carl Roth), 20 min RT	Life Technologies/Novex 40-2200	0,5 % Triton X-100	250 µg/ml	1:50
**Vimentin**	Methanol; -20 °C, 20 min	Dako Clone Vim 3B4	-	Not specified	1:50
**ACTA2**	Histofix 4 % (Carl Roth), 20 min RT	Abgent clone E184	0,5 % Triton X-100	Not specified	1:1000
**beta-Catenin**	Methanol; -20 °C, 20 min	Abcam ab6302	-	60 µg/ml	1:4000
**PCNA**	Methanol; -20 °C, 20 min	Abcam EPR3821	-	Not specified	1:50
**Desmin**	Histofix 4 % (Carl Roth), 20 min RT	Biorbyt, orb34054	0,5 % Triton X-100	50 µg/ml	1:50

Supplementary Table S4

Secondary Antibody	Description	Company	Dilution
**Goat anti-Rabbit IgG**	Dylight 594 conjugated highly cross absorbed	Thermo Scientific 35561	1:400
**Goat anti-Mouse IgG**	Dylight 488 multi species absorbed	AbD serotec STAR117D488	1:800

Supplementary Table S5

Primer	Forward (5'-3')	Reverse (5'-3')	Company
**Ephb2**	TGGCTACGGACCAAGTTCAT	TGGTGGCTGAGTCAAAGTCA	Sigma Aldrich
**KLF5**	TCACCTGAGAACTGGCCTCT	GGTCTGGTGGGAGCTGAATA	Sigma Aldrich
**ASCL2**	CTCGTCCCTGTCCTATCTGC	CTCGTCAAGCCTCCAAGTGT	Sigma Aldrich
**KLF4**	CAGGCACTACCGCAAACATA	TGTTGGGAACTTGACCATGA	Sigma Aldrich
**OLFM4**	GGCTGTGGATGAGAATGGAT	AAAGTACGGGTGGCGTACAG	Sigma Aldrich
**VIL1**	CTTCTTCGACGGTGACTGCT	CCGGCCCTTCAGAAAGTCAT	Sigma Aldrich
**LGR5**	CCTTGGCCCTGAACAAAATA	GCAGTGGGGAATTCATCAAG	Sigma Aldrich
